# The TREX2 3′→ 5′ Exonuclease Physically Interacts with DNA Polymerase δ and Increases Its Accuracy

**DOI:** 10.1100/tsw.2002.99

**Published:** 2002-02-01

**Authors:** Igor V. Shevelev, Kristijan Ramadan, Ulrich Hubscher

**Affiliations:** Institute of Veterinary Biochemistry and Molecular Biology, University of Zürich-Irchel, Winterthurerstrasse 190, CH-8057, Zürich, Switzerland; Department of Molecular and Radiation Biophysics, Petersburg Nuclear Physics Institute, Leningrad district, Gatchina, 188300, Russia

**Keywords:** isotonic and detergent, extracted pol δ, proofreading, TREX2 3′→ 5′ exonuclease

## Abstract

Proofreading function by the 3′→ 5′ exonuclease of DNA polymerase δ (pol δ) is consistent with the observation that deficiency of the associated exonuclease can lead to a strong mutation phenotype, high error rates during DNA replication, and ultimately cancer. We have isolated pol δ_d_ from isotonic (pol δ_i_) and detergent (pol δ_d_) calf thymus extracts. Pol δ_d_ had a 20-fold higher ratio of exonuclease to DNA polymerase than pol δ_i_. This was due to the physical association of the TREX2 exonuclease to pol δ_d_, which was missing from pol δ_i_. Pol δ_d_ was fivefold more accurate than pol δ_i_ under error-prone conditions (1 μM dGTP and 20 dATP, dCTP, and dTTP) in a M13mp2 DNA forward mutation assay, and fourfold more accurate in an M13mp2T90 reversion assay. Under error-free conditions (20 μM each of the four dNTPs), however, both polymerases showed equal fidelity. Our data suggested that autonomous 3′→ 5′ exonucleases, such as TREX2, through its association with pol I can guarantee high fidelity under difficult conditions in the cell (e.g., imbalance of dNTPs) and can add to the accuracy of the DNA replication machinery, thus preventing mutagenesis.

## INTRODUCTION

Eukaryotic DNA polymerases (pol) δ and ε have the ability to proofread replication errors by using their 3′→5′ exonuclease activities. Genetic analysis has shown that fidelity is essential for DNA replication, since mutations affecting the exonuclease activity pol δ and pol ε result in high mutation rates *in vivo*[1]. Moreover, transgenic mice with $exo^-$ mutated pol δ alleles showed a striking increase in cancer susceptibility within 12 months of age[2]. Nevertheless, several experimental results point to the possibility of additional proofreading by autonomous (not an inherent exonuclease of pol δ or pol ε) 3′→5′ exonucleases during DNA replication or repair. The base substitution error rate of pol δ has been shown to be 27-fold higher in the presence of proliferating cell nuclear antigen (PCNA)[3]. The inefficient excision of nucleotide analogues has been demonstrated for pol δ and ε[4]. The nucleotide substitution error rate for *Schizosaccaromyces pombe* pol δ holoenzyme (pol δ, replication factor C, and PCNA) was $5 \times 10^{-4}$ for T:G mispairs, two orders of magnitude lower than for *Escherichia coli* pol III holoenzyme at the same position[5]. Several mammalian 3′→5′ exonucleases such as Werner protein[6], p53[7], calf thymus[8], and rat liver autonomous 3′→5′ exonucleases[9] have been found to be associated with eukaryotic pol δ or other components of the replication fork. Finally, fidelity data of a human cell DNA replication complex suggested that additional fidelity factors are likely present during chromosomal replication *in vivo*[10,11]. In this paper we show that detergent extracted pol $\delta_d$ from calf thymus has a 20-times-higher exonuclease to DNA polymerase ratio than isotonic extracted pol $\delta_i$ (pol δ from isotonic extract). This higher ratio is due to the association of an additional 3′→5′ exonuclease, and the exonuclease was identified as TREX2[12].

As a consequence of this association pol $\delta_d$ (pol δ from detergent extract) was four to five times more accurate at error-prone conditions as determined in an *in vitro* forward mutation assay, suggesting that autonomous 3′→5′ exonuclease can proofread for pol δ.

## MATERIAL AND METHODS

Poly(dA)$_{1000-1500}$ and oligo(dT)$_{12-18}$ were purchased from Pharmacia and Sigma. Poly(dA)/oligo(dT) template (base ratio 10:1) was prepared as described by Weiser et al.[13]. PCNA was overexpressed in *E. coli* strain BL21 (DE3) harboring the expression plasmid pT7/PCNA and purified as described by Stucki et al.[14]. PCNA-dependent and -independent pol assays were carried out according to Weiser et al.[13]. 3′→5′ exonuclease was measured according to Belyakova et al.[15]. One unit of DNA polymerase activity corresponds to the incorporation of 1 nM total dNTPs into acid precipitate material in 60 min at 37ºC in a standard assay for pol δ[13]. One unit of exonuclease activity is defined as the amount of the enzyme causing the conversion of 10% 3′–end labeled ss DNA (0.2 μg, 10,000 cpm/μg) to acid soluble products in 60 min at 37ºC in 50 mM Tris-HCl pH 7.5, 1 mM DTT, and 5 mM $MgCl_2$. 3′→5′ exo:pol is the ratio of excised nucleotides (pmol) vs. those incorporated (pmol), when concentrations of limiting substrates being 0.1 Km include optimal conditions for each enzyme in separate parallel experiments.

Preparation of isotonic and detergent cell extracts: all isolation steps were performed at or near 0°C. The isotonic extract — (I): 300 ml buffer A (50 mM Tris pH 7.0, 10% (v/v) glycerol, 0.5 mM EDTA, 50 mM NaCl, 0.25 mM sucrose, 5 μg/ml pepstatin, 1 μg/ml leupeptin, 0.8 mM PMSF, and 1 mM DTT) — was added to 100 g calf thymus. The tissue was thawed, homogenized in a Sorvall Omnimixer, stirred gently for 2 h, and centrifuged at 25,000 × g for 45 min. The supernatant (supernatant I) was achieved. The pellet was again extracted as described above, and the supernatant (supernatant II) was mixed with supernatant I. From the mixed supernatants I and II, isotonic extract was obtained. The detergent extract — (D): the resulting pellet (40 g) (after supernatant II extraction) was resuspended in 120 ml of buffer B (50 mM Tris pH 7.0, 10% [v/v] glycerol, 0.5 mM EDTA, 50 mM NaCl, 1% Triton X-100, 5 μg/ml pepstatin, 1 μg/ml leupeptin, 0.8 mM PMSF, and 1 mM DTT), homogenized by 20 strokes in Dounce homogenizer, stirred for 2 h, and centrifuged at 12,000 × g for 45 min. The supernatant III was achieved. The pellet was again extracted on the same manner, and supernatant IV was achieved. Detergent extract was obtained from the mixed supernatants III and IV.

**TABLE 1**
**Characteristics of Calf Thymus pol $\delta_i$ and pol $\delta_d$[a]**

| Pol | (mg/ml) | Pol +PCNA (U/ml) | Pol -PCNA (U/ml) | Specific Activity (U/mg) | 3′→5′ Exo (U/ml) | Exo/Pol Ratio |
|---|---|---|---|---|---|---|
| pol $\delta_i$ | 0.1 | 64 | 1.8 | 640 | 204 | 3 |
| pol $\delta_d$ | 0.08 | 18 | 1.2 | 225 | 1100 | 61 |

[a]For details see Materials and Methods.

The subsequent purification steps for both pol $\delta_i$ and pol $\delta_d$ were described earlier[13], and some enzyme characteristics are shown in Table 1.

Pol δ from calf thymus isotonic extract was also purified with modified protocol that we used earlier. The isotonic extract was prepared on the same manner as before described. Fraction I (isotonic extract) was adsorbed to 60 ml phosphocellulose column (Whatman), and pol activity was eluted with linear gradient of 50–600 mM NaCl. Fractions eluted between 80–100 mM NaCl had pol activity with PCNA dependency (2-fold). These fractions were pooled to yield fraction II. Fraction II was loaded to 1 ml HiTrap heparin column (Amersham Pharmacia), and pol activity eluted with linear gradient of 50–1000 mM NaCl. Fractions eluted between 100–150 mM NaCl had PCNA dependency (5-fold). These fractions were pooled to yield fraction III. Fraction III was adsorbed on HiTrap Q column (Amersham Pharmacia), and pol activity eluted with linear gradient of 50–1000 mM NaCl. Fractions with 20-fold PCNA dependency (fraction IV) were eluted between 100–150 mM NaCl, containing mainly pol δ. Fraction IV was adsorbed on monoQ column (Amersham Pharmacia), and pol δ was eluted between 200–250 mM NaCl. The final fraction had 50-fold PCNA dependency.

Bacteriophage M13mp2 and *E. coli* strains CSH50, NR9099, and MC1061 were described by Kunkel[16]. Nonsense mutant M13 mp2T90 (point mutation G:T in the position +90 of *LacZ* gene) was selected after sequencing analysis of colorless mutants as mutation hot point in DNA M13mp2 forward mutation assay. The sequence of the 26 mer oligonucleotide was: 5′-cga tta agt tgg gta acg cca ggg tt-3′. Immunoprecipitation and immunobloting experiments were made according to Koundrioukoff et al.[17]. Chicken nonimmune IgY was used as a control for nonspecific binding in the immunoprecipitation experiment. The fusion human TREX2-MBP protein was expressed and purified as described by Mazur et al.[18] and used for producing chicken IgY according to Gassmann et al.[19]. Rabbit antisera against a peptide in N-terminal region of human pol δ (p125) was the gift of P. Fisher. Monoclonal antibody against human B-subunit of pol δ (p50) was kindly provided by H.-P. Nasheuer (Jena). Recombinant His-tagged p50 protein (B-subunit of pol δ) was overexpressed in *E. coli* and used as a positive control for the coimmunoprecipitation experiments.

## RESULTS AND DISCUSSION

In eukaryotes, spontaneous mutation rates have been estimated to be $10^{-10}$–$10^{-12}$ errors per base pair per generation. On the other hand, the principal elongation enzyme pol δ with its own 3′→5′ exonuclease activity site makes replication errors with probability of $10^{-5}$[20]. Our explanation for such an inaccuracy can be the loss of subunits of the eukaryotic pols that function during replication to improve the fidelity. Presumably, part of these errors could be eliminated by autonomous 3′→5′ exonucleases. Indeed, there is the immunochemical evidence for the complex formation between pol δ and some potent autonomous 3′→5′ exonucleases from calf thymus[21].

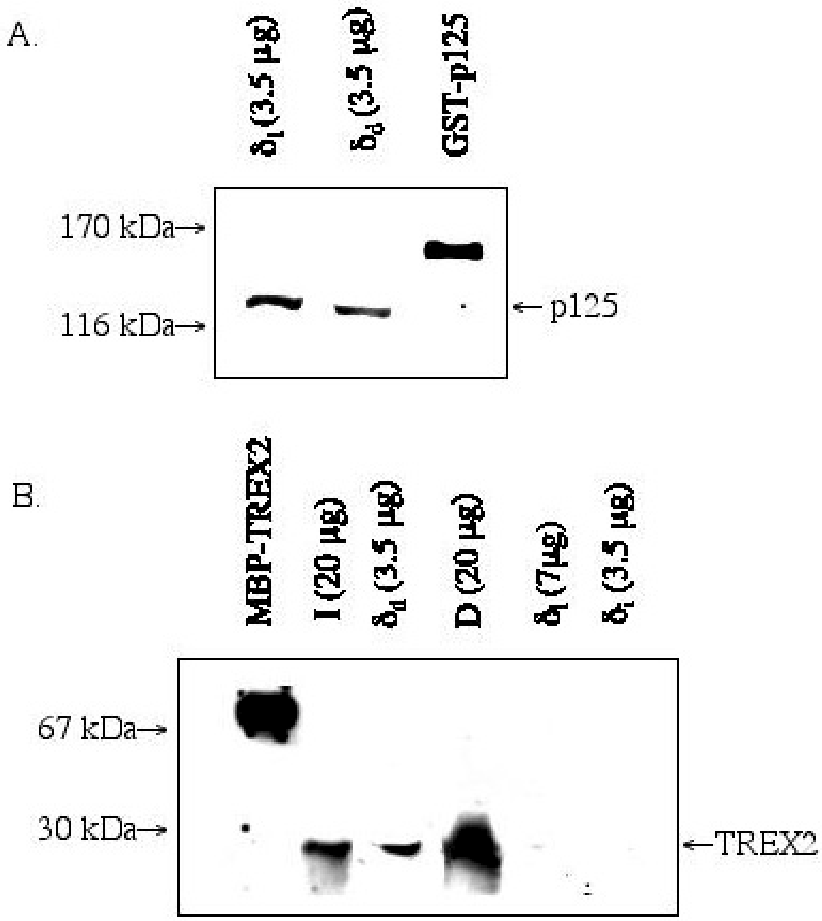

**FIGURE 1.** The TREX2 3′→5′ exonuclease copurifies with pol $\delta_d$. A: Western blot analysis of calf thymus pol $\delta_i$ and $\delta_d$ with polyclonal antibody against C-terminal part of catalytical subunit of pol δ (p125). B: Isotonic (I), detergent (D) calf thymus extracts pol $\delta_i$ and pol $\delta_d$ were loaded on 8% SDS-PAGE and transferred on nitrocellulose membrane. Polyclonal chicken antibody against TREX2 recognizes TREX2 exonuclease in both extracts and pol $\delta_d$.

Moreover, autonomous 3′→5′ exonucleases are responsible for 80% of the total 3′→5′ exonuclease activity in rat liver cells[9]. We showed, in our unpublished data, that additional 3′→5′ exonuclease such as DnaQ from *E. coli*, TREX1, TREX2, and Werner protein can enhance in 2- to 10-fold the accuracy of pol I of *E. coli*, pol δ, and ε from calf thymus under error-prone conditions in a M13mp2 forward mutation assay.

To approach this problem, we have monitored the purification of pol δ from isotonic and detergent calf thymus extracts. We obtained two pol δ preparations designated as pol $\delta_i$ and pol $\delta_d$ (Table1). Both pol δs were confirmed by immunobloting with antibodies against p125 (Fig. 1A) and p50 subunits (data not shown). The main difference between these two pol δs was a 20-times-higher ratio of 3′→5′ exonuclease to DNA polymerase. Both pol δs were free from endonuclease and 5′→3′ exonuclease activities (data not shown). Hoping to find a form of pol δ from isotonic extract with a more comparable exonuclease:DNA polymerase ratio, we have changed the purification protocol. However, we were able to purify pol δ by the modified protocol (see Material and Methods) than by Weiser et al.[13]. The other pol δ forms from isotonic extract had the same exonuclease:DNA polymerase ratio as pol $\delta_i$ (data not shown). These data suggested to us that pol $\delta_d$ might interact with an additional 3′→5′ exonuclease. The 3′→5′ exonuclease was identified in pol $\delta_d$ as TREX2 protein (Fig. 1B). The immunoprecipitation experiment also showed

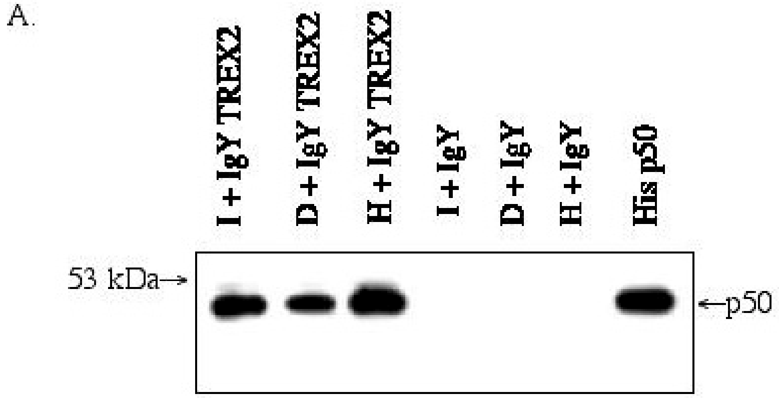

**FIGURE 2.** The TREX2 3′→5′ exonuclease physically interacts with pol δ. I, D (see Fig. 1B) and human (cell line Mg63, osteosarcoma) total cell extract (H) were immunoprecipitated with anti-TREX2 polyclonal antibody (IgY TREX2), or with chicken preimmune IgY as a control, and were analyzed by Western blot with rat monoclonal antibody against the small subunit of pol δ (p50). For this, a 12% SDS-PAGE and nitrocellulose membrane was used.

that TREX2 protein and pol δ form a complex in different prepared extracts (isotonic, detergent) (Fig. 2), indicating that TREX2 and pol δ interact. TREX2 also interacted with pol δ in human total cell extract suggesting that our finding is likely valid to any mammalian cells.

In order to understand a possible proofreading role of TREX2, we next used a forward mutation assay (Table 2). Pol $\delta_d$ showed the same mutation frequency as pol $\delta_i$ under error-free conditions (20 μM dNTPs). However, the TREX2 containing pol $\delta_d$ was about fivefold more accurate than pol $\delta_i$ under error-prone conditions (50-fold biased dGTP); 170 to 200 nucleotides were incorporated in each 250 target gap DNA for reactions of 2 h (data not shown). Next, the mutation frequency of base substitution was estimated for pol $\delta_i$ and pol $\delta_d$ (Table 3). To verify that in every case the nucleotides were incorporated in the nonsense codon (TAA, LacZ 90), an additional 26 mer oligonucleotide was hybridized to the gap molecules (the 3′-end of primer being located as three nucleotides from the point substitution T in the nonsense mutant instead of G in the M13mp2 at position +90 of LacZ gene). A fourfold higher fidelity for pol $\delta_d$ in comparison with pol $\delta_i$ was evident for base substitution under error-prone conditions.

**TABLE 2**
**Mutation Frequency of DNA Copied by pol $\delta_i$ and pol $\delta_d$ in a Forward Mutation Assay[b]**

| Pol | dNTPs | Total Plaques | Mutants Plaques | Mutation Frequency ($\times 10^{-4}$) |
|---|---|---|---|---|
| None | | 7,000 | 4 | 5.7 |
| pol $\delta_I$ | Equimolar | 7,000 | 8 | 11.4 |
| | Biased dGTP | 4,000 | 45 | 112.5 |
| pol $\delta_d$ | Equimolar | 9,000 | 10 | 11.1 |
| | Biased dGTP | 6,000 | 14 | 23.2 |

[b]Replication reactions were carried out for 2 h at 37ºC in 10 μl containing: 50 mM Tris-HCl pH 7.5, 1 mM DTT, 100 μg/ml BSA, 6 mM $MgCl_2$, 50 ng of gapped circular M13mp2 DNA, 20 μM each of dATP, dCTP, dGTP, and dTTP (equimolar) or additional 1 mM dGTP (biased dGTP), and pol $\delta_i$ (0.064 units) or pol $\delta_d$ (0.054 units).

**TABLE 3**
**Mutation Frequency of DNA Copied by pol $\delta_i$ and pol $\delta_d$ in a Reversion Mutation Assay[c]**

| Pol | dNTPs | Oligo[2] | Plaques Total | Mutant Plaques | Revertant Frequency ($\times 10^{-5}$) |
|---|---|---|---|---|---|
| None | - | - | 400, 000 | 6 | 1.5 |
| | | + | 520, 000 | 7 | 1.3 |
| pol $\delta_I$ | Equimolar | - | 320, 000 | 10 | 3.1 |
| | | + | 460, 000 | 14 | 3.0 |
| | Biased dGTP | - | 150, 000 | 48 | 32.0 |
| | | + | 220, 000 | 53 | 27.5 |
| pol $\delta_d$ | Equimolar | - | 280, 000 | 8 | 2.9 |
| | | + | 540, 000 | 17 | 3.1 |
| | Biased dGTP | - | 210, 000 | 18 | 8.6 |
| | | + | 290, 000 | 22 | 7.6 |

[c]Replication reactions were carried out for 2 h at 37ºC by using 50 ng gapped circular M13mp2T90 (sign – in oligo column), or for 10 min at 37ºC when a 26 mer oligonucleotide was hybridized in addition to the gapped circular M13mp2T90 DNA (sign + in oligo column). Other conditions were as in Table 2.

Our experiments for the proofreading function of TREX2 for pol δ have been demonstrated for the distributive synthesis of pol δ only. Next, it would be of interest to understand the proofreading of TREX2 for the processive synthesis of pol δ holoenzyme (pol δ, RF-C, RP-A, and PCNA).

## CONCLUSION

In conclusion we have identified from a detergent extract 3′→5′ exonuclease TREX2 associated with pol δ. The fact that this editing enzyme can enhance the fidelity of pol δ under difficult conditions strongly suggests that an autonomous exonuclease can proofread for pol δ. This might be important when pol δ has to dissociate from the DNA under genotoxic stress. As a consequence, this proofreading mechanism might help to minimize mutagenesis and consequently cancer propagation.

## ACKNOWLEDGEMENTS

We thank T.A. Kunkel for providing M13mp2 forward mutation assay components; F.W. Perrino for TREX2 plasmid constructs; R. Imhof and T. Tasara for DNA sequencing; E.A. Smirnova and N.V. Belyakova for assistance in producing and testing antibody; and V.M. Krutyakov for his consultation.

This work has been supported by EU TMR Grant (ERBMRXCT 970125) to IS and UH, by the Swiss National Science Foundation (Grant 31.61361.00) to KR and by the Kanton of Zürich to KR and UH, and by Russian Foundation for Basic Research (N 00-04-48867) to IS.
